# Federated Learning in Dentistry: Chances and Challenges

**DOI:** 10.1177/00220345221108953

**Published:** 2022-07-31

**Authors:** R. Rischke, L. Schneider, K. Müller, W. Samek, F. Schwendicke, J. Krois

**Affiliations:** 1 Department of Artificial Intelligence, Fraunhofer Heinrich Hertz Institute, Berlin, Germany; 2 Department of Oral Diagnostics, Digital Health and Health Services Research, Charité–Universitätsmedizin, Berlin, Germany; 3 ITU/WHO Focus Group on AI for Health, Topic Group Dental Diagnostics and Digital Dentistry, Geneva, Switzerland

**Keywords:** computer vision/convolutional neural networks, artificial intelligence, deep learning/machine learning, privacy, dental informatics/bioinformatics, mathematical modeling

## Abstract

Building performant and robust artificial intelligence (AI)–based applications for dentistry requires large and high-quality data sets, which usually reside in distributed data silos from multiple sources (e.g., different clinical institutes). Collaborative efforts are limited as privacy constraints forbid direct sharing across the borders of these data silos. Federated learning is a scalable and privacy-preserving framework for collaborative training of AI models without data sharing, where instead the knowledge is exchanged in form of wisdom learned from the data. This article aims at introducing the established concept of federated learning together with chances and challenges to foster collaboration on AI-based applications within the dental research community.

## Introduction

The potential of artificial intelligence (AI) to transform health care is vast. AI-based applications in dentistry may help in research, prevention, diagnostics, decision support, and automating routine tasks to facilitate treatment at low cost for more people, eventually allowing for personalized, predictive, preventive, and participatory dentistry (Schwendicke et al. 2020). However, particularly in health care, aspects related to data privacy and data sharing have been identified as hampering factors (Rieke et al. 2020). Especially, dentistry is affected by this, as patients can be identified from anonymized radiographs due to individual structures of tissues, tooth anatomy, and restoration status.

Hence, scalable methods for building AI that respect privacy constraints are required to unlock the full potential of AI-based applications in dentistry. A promising direction is the paradigm shift from centralized data-pooling for the development of AI to federated learning (FL) approaches. FL is closely related to deep learning, which is an AI subfield that aims at training neural networks to extract statistical patterns in given data to eventually make predictions on unseen data. During the so-called training phase, the neural network is iteratively and repeatedly exposed to training data, which consist of data points (e.g., images) with associated expert-based labels (e.g., “healthy” or “caries”). By minimizing the prediction error, the model learns, for instance, to distinguish healthy and decayed teeth in bitewing radiographs. A common approach for this training process is to first pool data and then train a model on these data, known as centralized learning. Such an approach, however, often lacks generalizability as the data pool stems from one or very few data sources (e.g., 1 or 2 contributing hospitals or research institutes). *Federated learning*, in contrast, enables multiple participants to collaboratively train AI models. FL widens the access to knowledge from many more and diverse data sources without sharing them directly. Instead, knowledge is exchanged in the form of trained AI models or their outputs (Rieke et al. 2020; Kairouz et al. 2021). This enables generalizability, while keeping the training data private, which is a well-known restriction for medical data analysis tasks. A central server (e.g., a trusted service provider) usually orchestrates the whole FL training process based on defined protocols.

**Figure 1.**
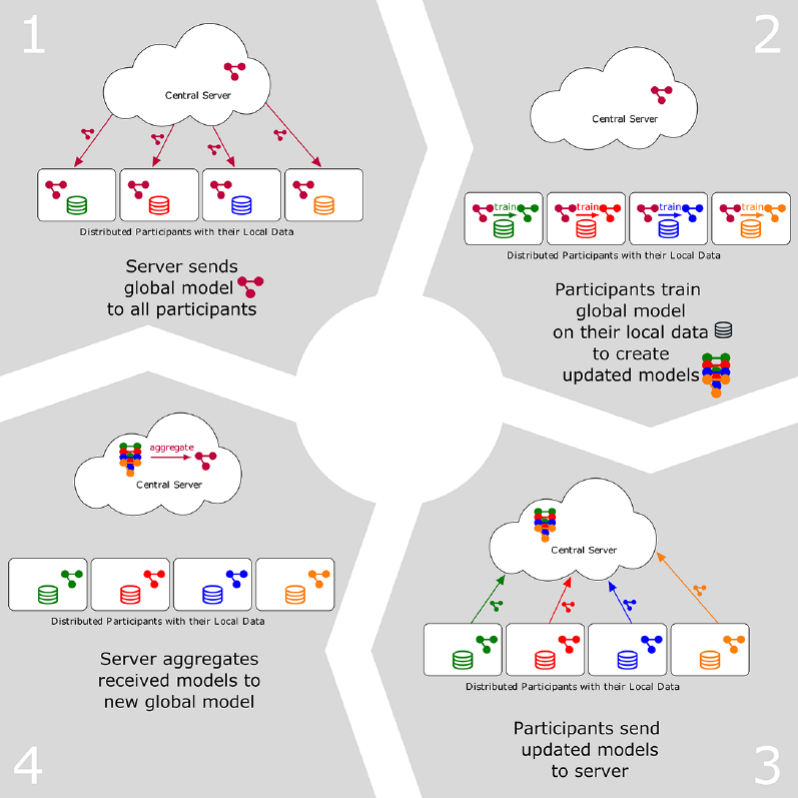
Algorithmic overview of the iterative federated averaging protocol with 4 steps: 1) server broadcasts artificial intelligence model to all participants; 2) each participant individually trains model on its local data to create an updated model; 3) participants send model updates back to central server; 4) server averages all model updates to aggregate them to a new global model for the next round.

One popular protocol for FL is *federated averaging* (*FedAvg*), introduced by McMahan et al. (2017), where all participants agree on an AI model (e.g., a certain neural network architecture), and each participant (e.g., research institute) trains its model on its local training data (e.g., collected bitewing radiographs with labels of “healthy” or “caries”). The updated models are then sent by the participants to the central server, which aggregates all received models to a new global model by averaging all model parameters. This global model carries knowledge originating from data of all participants and is broadcasted to all participants for the next round of this iterative training procedure, unless a certain stopping criterion is met. Figure 1 gives an overview of this communication protocol.

Another established algorithmic paradigm for FL is *federated distillation* (*FD*) (Jeong et al. 2018; Li and Wang 2019; Sattler, Korjakow, et al. 2021; Sattler, Marban, et al. 2021), where instead of model parameters (as in FedAvg), only model outputs for an unlabeled public data set are exchanged between participants and the central server. This approach allows participants to train heterogeneous model architectures but requires the existence of an appropriate public data set having similar distribution as the training data sets. Silva et al. (2018) published a public data set with 1,500 panoramic images, which may be used for employing FD in dentistry.

FL has already successfully fostered collaborative AI training without data sharing in different health care settings, especially for tasks in medical imaging (Roth et al. 2020; Sheller et al. 2020; Kaissis et al. 2021) and specifically also for international COVID-19 research (Raisaro et al. 2020; Dayan et al. 2021; Yang et al. 2021). The findings of these studies illustrate the value proposition of FL:

1. The performance of all local AI models regarding their predictive quality and generalizability can be improved by collaborative training (Roth et al. 2020; Dayan et al. 2021; Yang et al. 2021).
2. FL has the potential to achieve results of centralized learning (Sheller et al. 2020; Kaissis et al. 2021).
3. There are security mechanisms for preserving the privacy within FL (Raisaro et al. 2020; Kaissis et al. 2021).

As for dentistry, the above findings and insights apply likewise. Hence, we aim to introduce the FL concept, chances, current challenges, and potential to the dental research community. Embracing FL may foster collaboration and cultivate knowledge exchange, while respecting data privacy concerns, and overall improves AI-based applications in dentistry.

## Chances of Federated Learning in Dentistry

**Figure 2.**
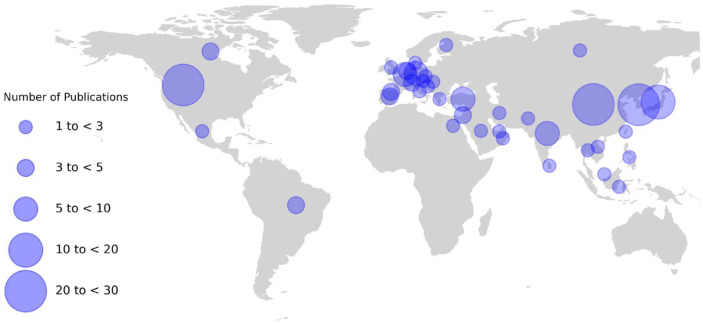
World map with artificial intelligence (AI)–related publications in dentistry (based on the first author’s affiliation) grouped by country. The data stem from a systematic review (unpublished) that screened the online repositories of Medline, IEEE, and arXiv for publications related to AI and dentistry that were published between 2015 and May 2021.

AI is an emerging field in dentistry. In 2020, over 240 AI-related publications were listed on PubMed, which among others predict the occurrence of caries lesions (Lee et al. 2018), periodontal bone loss (Krois et al. 2019), periodontally compromised teeth (Thanathornwong and Suebnukarn 2020), and apical lesions (Ekert et al. 2019). The number of institutes taking active part in this endeavor and their widespread geographic locations are represented in Figure 2. Training such AI-based systems requires large dental data sets, which ideally capture all possible anatomical structures and pathologies. More available data are likely to improve the predictive performance of models, and with more diversity of samples, models are likely to generalize better across different data sources. However, the procurement of such data is difficult as it takes a considerable amount of time, effort, and financial resources to collect and store the data as well as to establish a ground truth. Especially, the latter aspect is often a limiting factor for the size of data sets in dentistry, as most use-cases are based on segmentation or detection tasks, which are generally more time-consuming in ground truth generation than classification tasks. As this holds for many applications, FL is also better understood for classification tasks than segmentation or detection, which necessitates further research in this direction.

In addition, dental data are considered highly sensitive, and data sharing is regulated to preserve the privacy of patients, which restricts the publication of data sets (Van Panhuis et al. 2014). To address this, several techniques for deidentification have been proposed, such as removing patient-specific information (i.e., age or date of birth). Yet, it has been shown that this is not sufficient to protect patients’ privacy (Rocher et al. 2019). Especially dental images and radiographs bear a high risk for reidentification. Structures of hard and soft tissue as well as teeth anatomy and dental restorations are unique to individuals, which make radiographs highly relevant for forensic odontology. In this field, reidentification is highly desired: a person, often an unknown deceased individual, may be identified by comparing ante- and postmortem dental radiographs. For example, after the tsunami disaster in 2004, 79% of deceased individuals were identified based on intraoral radiography alone (James et al. 2005). For general data sharing between institutes, these reidentification capabilities of forensic odontology highlight the risk of privacy sensitivity and possible privacy breaches.

To enable joint data-driven research between dental institutes, FL addresses these privacy concerns with its privacy-by-design approach of avoiding raw data exchanges and instead sharing the wisdom learnt from the data, as described in the previous section. A typical setting for dentistry is *cross-silo* FL (Kairouz et al. 2021). Thereby, a few participants (e.g., 2–100 hospitals) have access to large data silos that they cannot share directly with each other due to the described privacy concerns.

In addition to the privacy aspects, FL also offers a solution to data and model ownership concerns present in centralized learning, as in FL data never leave their source and the AI model is trained by all participants. This can be a security advantage, as in FL, there is not only a single institute that an attacker can concentrate on to tamper the training process or get access to private information.

Overall, FL offers great potential to accelerate advances in AI for dentistry. There are already many distinguished gravitational centers of AI research in dentistry (see Fig. 2), so that the provision of a technology that connects them may result in a tremendous development boost for the community. Each research group most likely has access to large amounts of siloed data of varying modalities and may be willing to contribute to collaborative training of dental AI. Dentistry by nature offers beyond the already existing AI research centers many potential FL collaborators, as it is common to store data from small dental offices at large research institutions, which potentially have the required resources to overcome initial investments to join a federation.

## Coping with Challenges for FL in Dentistry

Despite all the chances of FL for dentistry, there are also challenges that require appropriate solutions to unlock the potential of FL. First, FL requires AI preknowledge of the participants, since they agree on a certain model architecture (in FedAvg) or train on their individual architecture (in FD). Performing an architecture search (Elsken et al. 2019) within FL is computationally very expensive and thus should happen before the joint training phase on the local data to find an appropriate starting model for the considered learning task. Similarly, the tuning process of parameters as well as monitoring and debugging of FL systems are more difficult without direct access to data. Within FL, all participants should have appropriate local computational resources to join the federation, which may be a high initial investment for new participants. Furthermore, these learning resources are costly in maintenance and require higher coordination and effort for deployment than centralized systems.

Second, the participants’ data silos are often statistically heterogeneous by nature due to different medical standards as well as social and economic determinants, also known as data set shift (Quionero-Candela et al. 2009). More formally, the whole training data are usually not distributed in an *iid* (independent and identically distributed) fashion among the participants, which would be fulfilled in an ideal FL setting. Data heterogeneity (“non-iid-ness”), however, usually has negative effects on the final model performance in FL and is also a cause of the performance gap to centralized learning (Li et al. 2018; Zhao et al. 2018; Hsu et al. 2019; Kairouz et al. 2021; Sattler, Korjakow, et al. 2021; Sattler, Müller, and Samek 2021). Data heterogeneity in dentistry may be caused by a covariate shift (e.g., different x-ray machines used for data acquisition), prior probability shift (e.g., more implants in developed countries), or unbalancedness (e.g., some regions collect more data), as characterized by Kairouz et al. (2021).

FL applications usually must cope with a mixture of these types, especially when applied at an international scale. However, tasks in dentistry are usually less driven by personal preferences but rather by defined standards (e.g., when considering caries classification). This is crucial for a feasible FL task, as shown by other success stories in medical imaging outlined in the first section.

There are different strategies for coping with data heterogeneity in FL (see, e.g., Kairouz et al. 2021). General approaches are data set augmentation by publicly available data or sharing some data between participants (Zhao et al. 2018) to make the data silos statistically more homogeneous as well as modifying existing FL algorithms together with their hyperparameters for more effective and efficient training in the presence of data heterogeneity (Li et al. 2018; Li and Wang 2019; Karimireddy et al. 2020; Sattler, Korjakow, et al. 2021). If the assumption that the participants share a similar probability distribution is not fulfilled, meaning that training a single model satisfying all participants is not possible, then more specific strategies are required such as personalization of the global model (Wang et al. 2019), multitask learning (Smith et al. 2017), participant clustering (Sattler, Müller, and Samek 2021), and meta-learning (Khodak et al. 2019).

Third, although FL is a privacy-by-design approach and has, due to its decentralized nature, a lower security risk than centralized learning, vanilla FL protocols without further protection mechanisms have been shown to be vulnerable to certain privacy attacks (Kairouz et al. 2021; Mothukuri et al. 2021). While attacks in centralized learning usually focus on compromising the central server to leak data, privacy attacks in FL rather aim at the reconstruction of the participants’ local training data or tampering the training process to leak private information through model inversion attacks (Fredrikson et al. 2015), membership inference attacks (Shokri et al. 2017), or Generative Adversarial Network (GAN) attacks (Hitaj et al. 2017). Thus, privacy within FL systems often also depends on the system’s security. Hence, setting up an FL system that is based on trust and secured by cryptography (e.g., secure multiparty computation or homomorphic encryption) is a common approach to preserve privacy (Kairouz et al. 2021; Kaissis et al. 2021; Mothukuri et al. 2021). Another established concept for privacy preservation within FL is differential privacy (Dwork and Roth 2014), where carefully chosen noise is added to the model parameters or model outputs to obfuscate them before release so that it is hard to reconstruct individual training samples. Differential privacy provides a mathematically provable guarantee of privacy but often creates a privacy versus performance trade-off.

Fourth, the value propositions of FL in the first section might already motivate owners of data silos to join FL efforts. Nonetheless, FL requires an investment in local computing resources (including maintenance and coordination) and is based on trust among the participants. Both investments should pay off fairly to motivate participation. Designing mechanisms for FL that incentivize truthful participation and fairness is an interesting open research question (Donahue and Kleinberg 2021; Shi et al. 2021; Witt et al. 2021).

## Call for Action for Dental Researchers

The authors encourage researchers to join their forces to improve AI-based applications in dentistry through collaborative FL initiatives. To get started, interested parties should get familiar with the FL life cycle and training process as, for instance, described in Kairouz et al. (2021). Based on a solid understanding of the theoretical concepts, one may decide on the data modality, the task to be solved, and potential collaborators for the FL initiative. The topic group “Dental Diagnostics and Digital Dentistry” (TG-Dental) within ITU/WHO Focus Group Artificial Intelligence for Health (FG-AI4H) provides a point of contact to find collaborators. Once the general conditions are defined, all participants must agree on certain settings of the FL process. This includes a decision on the collaboratively trained model architecture. A consensus might be reached by simulations of the FL process, which is not necessary if a well-performing model architecture is already known. Alternatively, participants may use the FD protocol, allowing each participant to train individual architectures. As discussed in the previous section, setting up an FL system can be challenging in practice given the constraints in health care. Thus, the discussed aspects of data heterogeneity, privacy, security, and incentives should be considered already during the initial design phase. Finally, the implementation of the FL training pipelines may be supported by frameworks such as Flower (https://flower.dev/), PySyft (https://github.com/OpenMined/PySyft), NVIDIA Clara (https://developer.nvidia.com/clara-medical-imaging), or TensorFlow Federated (https://www.tensorflow.org/federated).

## Conclusion

FL is an established, scalable, and privacy-preserving concept for collaborative AI training without data sharing. Its value propositions have been successfully verified in different medical domains. Although challenges remain to be solved, dentistry should be among the early adopters to use the potential that lies in this concept. This in turn may foster digitalization and standardization efforts in dentistry to address some of the discussed challenges.

## Author Contributions

R. Rischke, L. Schneider, contributed to conception, design, data analysis, and interpretation, drafted and critically revised the manuscript; K. Müller, W. Samek, contributed to conception and design, critically revised the manuscript; F. Schwendicke, contributed to conception, design, data analysis, and interpretation, critically revised the manuscript; J. Krois, contributed to conception, design and data analysis, drafted and critically revised the manuscript. All authors gave final approval and agree to be accountable for all aspects of the work.
